# Characterization of linezolid- and methicillin-resistant coagulase-negative *staphylococci* in a tertiary hospital in China

**DOI:** 10.1186/s12879-024-09376-z

**Published:** 2024-05-10

**Authors:** Cailin Liu, Jing Yu, Chunguang Chen, Xiaogai Li, Yafei Ye, Yani Dong, Xinxin Ying, Haijun Li, Wanhai Wang

**Affiliations:** 1 Department of Clinical Laboratory, The First Affiliated Hospital of Zhengzhou University, No.1 Jianshe East Road, Erqi District, Zhengzhou, Henan Province 450052 China; 2 Key Clinical Laboratory of Henan Province, Zhengzhou, China; 3 Department of Clinical Laboratory, The Third Affiliated Hospital of Zhengzhou University, Zhengzhou, China; 4 Department of Clinical Laboratory, The Sixth People’s Hospital of Zhengzhou City, Zhengzhou, China; 5 Department of Clinical Laboratory, Yichuan People’s Hospital, Zhengzhou, China; 6 Department of Clinical Laboratory, Luohe Sixth People’s Hospital, Zhengzhou, China

**Keywords:** Coagulase-negative staphylococci, Linezolid-resistant, G2576T mutation, *cfr* gene

## Abstract

**Background:**

Recently, linezolid-resistant staphylococci have become an emerging problem worldwide. Understanding the mechanisms of resistance, molecular epidemiology and transmission of linezolid-resistant CoNS in hospitals is very important.

**Methods:**

The antimicrobial susceptibilities of all isolates were determined by the microdilution method. The resistance mechanisms and molecular characteristics of the strains were determined using whole-genome sequencing and PCR.

**Results:**

All the strains were resistant to oxacillin and carried the *mecA* gene; 13 patients (36.1%) had prior linezolid exposure. Most *S. epidermidis* and *S. hominis* isolates were ST22 and ST1, respectively. MLST typing and evolutionary analysis indicated most linezolid-resistant CoNS strains were genetically related. In this study, we revealed that distinct CoNS strains have different mechanisms of linezolid resistance. Among ST22-type *S. epidermidis*, acquisition of the T2504A and C2534T mutations in the V domain of the 23 S rRNA gene, as well as mutations in the ribosomal proteins L3 (L101V, G152D, and D159Y) and L4 (N158S), were linked to the development of linezolid resistance. In *S. cohnii* isolates, *cfr*, S158Y and D159Y mutations in the ribosomal protein L3 were detected. Additionally, emergence of the G2576T mutation and the *cfr* gene were major causes of linezolid resistance in *S. hominis* isolates. The *cfr* gene, G2576T and C2104T mutations, M156T change in L3 protein, and I188S change in L4 protein were found in *S. capiti*s isolates.

**Conclusion:**

The emergence of linezolid-resistant CoNS in the environment is concerning because it involves clonal dissemination and frequently coexists with various drug resistance mechanisms.

## Background

Coagulase-negative *staphylococci* (CoNS) have become important pathogens in patients with health care-associated infections caused by indwelling medical devices or immunocompromised patients [1, 2]. According to the monitoring data from the China Antimicrobial Resistance Surveillance System (CARSS, www.carss.cn/), the frequency of methicillin-resistant coagulase-negative *staphylococci* (MRCoNS) was extremely high, and the prevalence of MRCoNS was 74.5% in 2021.

Linezolid is an oxazolidinone antibiotic that is a last-resort antibiotic for the treatment of serious infections caused by gram-positive bacteria, including drug resistant organisms, such as methicillin-resistant *staphylococci* and vancomycin-resistant *enterococci* [3, 4]. The first clinical isolates of linezolid-resistant *staphylococci* and *enterococci* were reported in 2001 [5, 6]. Since then, linezolid-resistant organisms have been sporadically reported worldwide [7–10]. However, the detection rate of linezolid-resistant coagulase-negative *staphylococci* has progressively increased in recent years in China.

Point mutation in the domain V region of the 23 S rRNA gene was the most common mechanism of linezolid resistance, and the most frequent mutation was G2576T. In addition, T2500A, T2604C, C2532T, C2551T, G2603T, G2614T, C2190T, and G2447T mutations have been reported [11–13]. Furthermore, other mechanisms of linezolid resistance in *staphylococci* have also been reported, such as the presence of the *cfr* gene, which encodes an rRNA methyltransferase, and mutations in the ribosomal proteins L3, L4 and L22 [14–16].

In China, the prevalence of linezolid-resistant *staphylococci* has gradually increased in recent years; however, very few studies have investigated linezolid resistance. In this study, the clinical characteristics, antibiotic susceptibility, and resistance mechanisms of linezolid-resistant MRCoNS isolates that were recovered from 2019 to 2023 in a Chinese tertiary hospital were investigated.

## Materials and methods

### Bacterial isolates

Thirty-seven linezolid-resistant MRCoNS isolates, including thirteen *Staphylococcus capitis* isolates, nine *Staphylococcus hominis* isolates, eight *Staphylococcus epidermidis* isolates, six *Staphylococcus cohnii* isolates and one *Staphylococcus haemolyticus* isolate, were collected from December 2019 to March 2023 in a large tertiary teaching hospital (The First Affiliated Hospital of Zhengzhou University, Zhengzhou, with 8,000 beds located in east-central China). Two strains were isolated from pleural fluid, two from catheter tips, one from abdominal dropsy fluid, and one from cerebrospinal fluid; the remaining strains were recovered from blood cultures. Thirty-seven linezolid-resistant MRCoNS isolates were isolated from 36 patients, 25 of whom were male and 11 of whom were female. Linezolid-resistant *S. epidermidis*-5 and *S. capitis*-33 were isolated from the same patient, a 76-year-old man who was admitted to the respiratory intensive care unit. In addition, all patients were administered antibiotics, and 13 patients (36.1%) had received prior linezolid treatment. The characteristics of isolates and associated clinical data are listed in Table 1. Identification of the organisms was carried out using a VITEK®2 Compact system and VITEK® MS (bioMérieux, Marcy-l’Étoile, France).

**Table 1:** Clinical characteristics of patients with linezolid-resistant MRCoNS

Isolate	Collection date(mm/dd/yy)	Sex	Age(y)	Ward	Source	Antibiotic exposure	Clinical diagnosis	outcome
*S.epidermidis*-1	02-18-22	M	67	RICU	pf	BIA, LVX, TGC	Severe pneumonia	death
*S.epidermidis*-2	11-28-20	F	74	neurosurgery	sf	CAZ, VAN	Postoperative recurrence of meningioma	Discharge
*S.epidermidis*-3	07-06-21	M	63	ICU	bl	ATM, LNZ, VAN	Sepsis	Discharge
*S.epidermidis-*4	04-05-22	F	48	ICU	bl	SCF,TEC	Cerebral hemorrhage	death
*S.epidermidis*-5	03-18-22	M	76	RICU	pf	LNZ, BIA, DOX, LVX, TGC	Sepsis	Discharge
*S.epidermidis*-6	07-14-22	M	59	ICU	bl	SCF, VAN	Sepsis	Discharge
*S.epidermidis*-7	07-30-22	F	54	ICU	bl	SCF	Malignant tumor of esophagus	Discharge
*S.epidermidis*-8	07-16-22	M	53	ICU	bl	MEM	Multiple organ failure	death
*S.cohnii-*9	01-13-21	F	45	ICU	bl	LNZ,TEC, BIA	Pulmonary malignant tumor	Discharge
*S.cohnii*-10	07-29-22	M	69	RICU	bl	CXM	Pericarditis	Discharge
*S.cohnii*-11	08-16-22	F	28	ICU	bl	CAZ, MOX	Pneumonia	Discharge
*S.cohnii*-12	07-28-22	M	68	ICU	bl	SCF, BIA, LNZ, IPM	Cirrhosis	Discharge
*S.cohnii*-13	06-20-21	M	52	ICU	bl	CXM,CNX	Malignant tumor of the right ureter	Discharge
*S.cohnii*-14	03-21-23	M	14	EICU	bl	SCF, LNZ	Drug poisoning	Discharge
*S.hominis*-15	11-30-20	F	62	ICU	bl	CNX	Gastrointestinal hemorrhage	death
*S.hominis*-16	01-13-21	M	61	AICU	bl	SCF, LNZ	liver transplantation	Discharge
*S.hominis*-17	04-23-21	M	68	RICU	bl	CXM, SCF, IPM, TEC	Malignant neoplasm of rectum	Discharge
*S.hominis*-18	05-26-21	F	60	RICU	bl	BIA, IPM, SCF, TGC, TEC	Septic shock	death
*S.hominis*-19	04-09-21	M	53	RICU	bl	IPM, TEC	Septic shock	death
*S.hominis*-20	08-14-22	M	88	emergency surgery	bl	SCF	Gastric perforation	Discharge
*S.hominis*-21	08-13-22	F	70	ICU	bl	BIA, SCF, VAN	Lumbar spinal stenosis	Discharge
*S.hominis*-22	02-17-23	M	76	EICU	bl	SCF	Pneumonia	Discharge
*S.hominis*-23	03-15-23	M	76	AICU	bl	LVX, TZP, IPM, TEC, MEM	Malignant neoplasm of rectum	Discharge
*S. capitis-*24	12-31-19	M	37	ICU	bl	CXM, TZP, LNZ, TGC	Acute disseminated encephalomyelitis	Discharge
*S. capitis*-25	01-17-20	M	64	ICU	bl	TZP,TGC, GEN, CAZ, MEM, LNZ	Cerebral hemorrhage	Discharge
*S. capitis*-26	10-31-20	F	75	ICU	bl	TZP	Cerebral infarction	death
*S. capitis*-27	04-21-20	M	41	ICU	bl	MOX, LNZ, TZP, LVX	Cerebral hemorrhage	Discharge
*S. capitis*-28	03-26-21	M	48	RICU	bl	MOX, LNZ, BIA	Cerebral hemorrhage	Discharge
*S. capitis*-29	02-16-21	M	65	Respiratory	ab	IPM, MEM, LNZ	Primary hepatocellular carcinoma	Discharge
*S. capitis-*30	01-12-21	M	62	ICU	bl	MOX, BIA	Cardiac arrest	death
*S. capitis*-31	11-14-20	M	57	RICU	bl	BIA, LNZ	Severe pneumonia	Discharge
*S. capitis-*32	04-01-22	F	73	ICU	cs	IPM, TZP	Atrial fibrillation	Discharge
*S. capitis*-33	02-18-22	M	76	RICU	bl	LNZ, BIA, LVX, TGC	Sepsis	Discharge
*S. capitis*-34	02-22-22	M	10	ICU	bl	MEM, POL	Cerebral hemorrhage	death
*S. capitis*-35	10-17-22	M	72	ICU	bl	TZP	Cerebral hemorrhage	Discharge
*S. capitis*-36	03-11-23	F	68	ICU	cs	LNZ	Subarachnoid hemorrhage	Discharge
*S. haemolyticus*-37	02-12-23	M	88	RICU	bl	BIA	Severe pneumonia	Discharge

ICU, intensive care unit; RICU, respiratory intensive care unit; EICU, emergency intensive care unit; AICU: anesthesia intensive care unit. pf, pleural fluid; ab, abdominal dropsy; sf, cerebrospinal fluid; bl, blood; cs, catheter tip. BIA, biapenem; LVX, levofloxacin; TGC,tigecycline; CAZ, ceftazidime; VAN, vancomycin; ATM, aztreonam; LNZ, linezolid; SCF, cefoperazone/sulbactam; TEC, teicoplanin; DOX, doxycycline; MEM, meropenem; IPM, imipenem; CXM, cefuroxime; CNX, cefminox; TZP, piperacillin/tazobactam; GEN, gentamicin; MOX,moxalactam; POL, polymyxin B

### Antimicrobial susceptibility testing

The antimicrobial susceptibilities of all the isolates were determined using the microdilution method. *S. aureus* ATCC 29213 was used as a for quality control strain for susceptibility testing. The breakpoint for tigecycline treatment was interpreted according to the European Committee on Antimicrobial Susceptibility Testing Guidelines (http://www.eucast.org/clinical_breakpoints/). The results of susceptibility testing for linezolid, penicillin, oxacillin, chloramphenicol, clindamycin, erythromycin, gentamicin, trimethoprim/sulfamethoxazole, ciprofloxacin, levofloxacin, rifampin, tetracycline, vancomycin and teicoplanin were interpreted according to the Clinical and Laboratory Standards Institute (CLSI) M100-S33 breakpoints.

### Genomic DNA extraction and whole-genome sequencing

Genomic DNA was extracted using the Puregene Yeast/Bacteria Kit (QIAGEN) according to the manufacturer’s instructions for gram-positive bacteria. Whole-genome sequencing (WGS) was performed using the Illumina HiSeq PE150 platform (Novogene Bioinformatics Technology Co., Ltd., Beijing, China). The acquired antibiotic resistance genes (ARGs) carried by the isolates were analyzed using KmerResistance v 2.2 [17] with raw reads. The results were reported for reads with ≥ 90% nucleotide identity, ≥ 90% coverage of the query, and a sequence depth of ≥ 10×. The raw reads were assembled into scaffolds using SPAdes v 3.13.1 [18]. Multilocus sequence typing (MLST) of the isolates was performed using MLST 2.0 (https://cge.food.dtu.dk/services/MLST/) and further validated with PubMLST (https://pubmlst.org) for *S. epidermidis*, *S. hominis*, and *S. haemolyticus*. Additionally, the phylogenetic tree of *S. capitis* and *S. cohnii* isolates was constructed using BacWGSTdb 2.0 (http://bacdb.cn/BacWGSTdb/index.php).

### Molecular detection of resistance genes and mutations

Domain V of the 23 S rRNA gene and *cfr* gene was amplified and sequenced as previously described [19, 20], and the *rplC*, *rplD*, and *rplV* genes, which encode the ribosomal proteins L3, L4 and L22, respectively, were tested using previously described conditions and primers [10, 21].

## Results

### Antimicrobial susceptibility

All 37 isolates displayed varying degrees of resistance to penicillin, oxacillin, and linezolid, and the *mecA* gene was detected in all isolates. Most of the isolates were resistant to chloramphenicol, levofloxacin, ciprofloxacin, clindamycin, erythromycin, and gentamicin, with resistance rates of 94.6% (35/37), 89.2% (33/37), 89.2% (33/37), 91.9% (34/37), 67.6% (25/37), and 54.1% (20/37), respectively. Interestingly, only 1 of the 8 *S. epidermidis* isolates was resistant to erythromycin; however, all thirteen *S. capitis* strains were resistant to erythromycin. The resistance rates of the linezolid-resistant MRCoNS strains to trimethoprim/sulfamethoxazole, tetracycline and rifampicin were 27.0% (10/37), 16.2% (6/37), and 10.8% (4/37), respectively; however, six *S. cohnii* isolates were sensitive to trimethoprim/sulfamethoxazole. No resistance to tigecycline, vancomycin or teicoplanin was detected(Table 2).

**Table 2:** Antimicrobial susceptibility of linezolid-resistant MRCoNS

Isolate	**MIC(µg/mL)**															
	**LNZ**	PEN	OXA	**CHL**	CLI	ERY	Inducible Clindamycin Resistance	GEN	SXT	CIP	LVX	RIF	TCY	TGC	VAN	TEC
*S.epidermidis*-1	> 128	2	>=8	>=32	>=4	0.5	neg	>=32	1/19	>=4	>=8	<= 0.5	<=2	0.25	1	0.5
*S.epidermidis*-2	> 128	1	>=8	>=32	>=4	0.5	neg	>=32	4/76	>=4	>=8	<= 0.5	<=2	0.12	1	2
*S.epidermidis*-3	> 128	0.25	>=8	>=32	>=4	0.5	neg	>=32	1/19	>=4	>=8	4	<=2	0.25	1	4
*S.epidermidis-*4	> 128	0.25	>=8	>=32	>=4	0.5	neg	>=32	1/19	>=4	>=8	>=8	<=2	0.12	1	4
*S.epidermidis*-5	> 128	0.25	>=8	>=32	>=4	0.5	neg	16	<=0.5/9.5	>=4	>=8	<= 0.5	<=2	0.25	1	2
*S.epidermidis*-6	> 128	2	>=8	>=32	>=4	0.5	neg	16	1/19	>=4	>=8	2	<=2	0.25	1	2
*S.epidermidis*-7	8	>=16	>=8	>=32	>=4	>=8	pos	>=32	4/76	>=4	>=8	<= 0.5	<=2	0.25	1	1
*S.epidermidis*-8	> 128	0.25	>=8	>=32	>=4	0.5	neg	16	1/19	>=4	>=8	<= 0.5	<=2	0.25	1	4
*S.cohnii-*9	64	2	>=8	>=32	>=4	>=8	pos	8	<=0.5/9.5	>=4	>=8	<= 0.5	>=32	0.25	1	2
*S.cohnii*-10	8	2	>=8	8	>=4	>=8	pos	4	<=0.5/9.5	>=4	>=8	<= 0.5	<=2	0.25	1	2
*S.cohnii*-11	32	2	>=8	>=32	>=4	>=8	pos	>=32	<=0.5/9.5	>=4	>=8	<= 0.5	<=2	0.25	1	2
*S.cohnii*-12	128	4	>=8	>=32	>=4	>=8	pos	>=32	<=0.5/9.5	>=4	>=8	<= 0.5	>=32	0.25	1	2
*S.cohnii*-13	128	2	>=8	>=32	>=4	>=8	pos	>=32	<=0.5/9.5	>=4	>=8	<= 0.5	>=32	0.25	1	1
*S.cohnii*-14	128	>=16	>=8	>=32	>=4	<=0.25	neg	4	<=0.5/9.5	>=4	>=8	<= 0.5	>=32	0.25	1	0.5
*S.hominis*-15	16	>=16	>=8	>=32	>=4	>=8	pos	>=32	>=8/152	>=4	>=8	>=8	>=32	0.25	0.5	0.5
*S.hominis*-16	32	4	>=8	>=32	1	1	neg	4	4/76	2	2	<= 0.5	<=2	0.12	1	8
*S.hominis*-17	16	0.25	4	>=32	1	0.5	neg	4	4/76	2	2	<= 0.5	<=2	0.25	0.5	4
*S.hominis*-18	32	1	>=8	>=32	1	0.5	neg	<=2	4/76	2	2	<= 0.5	<=2	0.12	1	8
*S.hominis*-19	16	>=16	>=8	>=32	>=4	>=8	pos	16	>=8/152	>=4	>=8	<= 0.5	<=2	0.12	0.5	0.25
*S.hominis*-20	16	>=16	>=8	16	>=4	>=8	pos	8	4/76	>=4	>=8	<= 0.5	<=2	0.12	1	0.25
*S.hominis*-21	8	>=16	>=8	>=32	>=4	>=8	neg	8	<=0.5/9.5	2	1	<= 0.5	<=2	0.12	0.5	< 0.125
*S.hominis*-22	8	>=16	>=8	>=32	>=4	4	neg	>=32	<=0.5/9.5	>=4	4	<= 0.5	<=2	0.12	0.5	< 0.125
*S.hominis*-23	> 128	>=16	>=8	>=32	>=4	>=8	pos	8	<=0.5/9.5	>=4	>=8	1	>=32	0.25	2	1
*S. capitis-*24	> 128	>=16	>=8	>=32	>=4	>=8	pos	<=2	<=0.5/9.5	>=4	>=8	<= 0.5	<=2	0.25	0.5	0.25
*S. capitis*-25	> 128	>=16	>=8	>=32	>=4	>=8	pos	<=2	<=0.5/9.5	>=4	>=8	<= 0.5	<=2	0.25	0.5	0.25
*S. capitis*-26	32	>=16	>=8	>=32	1	>=8	pos	<=2	1/19	>=4	>=8	<= 0.5	4	0.25	0.5	0.25
*S. capitis*-27	32	>=16	>=8	>=32	1	>=8	pos	>16	1/19	>=4	>=8	<= 0.5	4	0.25	0.5	0.25
*S. capitis*-28	> 128	>=16	>=8	>=32	>=4	>=8	pos	<=2	<=0.5/9.5	>=4	>=8	<= 0.5	4	0.25	1	0.25
*S. capitis*-29	> 128	>=16	>=8	>=32	>=4	>=8	pos	<=2	<=0.5/9.5	>=4	>=8	1	<=2	0.25	1	0.25
*S. capitis-*30	32	>=16	>=8	>=32	1	>=8	pos	>=32	2/38	>=4	>=8	2	4	0.25	0.5	0.25
*S. capitis*-31	> 128	>=16	>=8	>=32	>=4	>=8	pos	4	<=0.5/9.5	>=4	>=8	<= 0.5	4	0.5	0.5	0.25
*S. capitis-*32	32	>=16	>=8	>=32	1	>=8	pos	>=32	>=8/152	>=4	>=8	<= 0.5	4	0.25	1	0.25
*S. capitis*-33	> 128	>=16	>=8	>=32	>=4	>=8	pos	<=2	1/19	>=4	>=8	<= 0.5	4	0.25	0.5	0.25
*S. capitis*-34	> 128	>=16	>=8	>=32	>=4	>=8	pos	<=2	<=0.5/9.5	>=4	>=8	<= 0.5	4	0.25	0.5	0.25
*S. capitis*-35	32	>=16	>=8	>=32	1	>=8	pos	>=32	1/19	>=4	>=8	<= 0.5	4	0.25	0.5	0.25
*S. capitis*-36	32	>=16	>=8	>=32	>=4	>=8	pos	>=32	<=0.5/9.5	>=4	>=8	<= 0.5	4	0.5	1	0.25
*S. haemolyticus*-37	> 128	>=16	>=8	>=32	>=4	>=8	pos	>=32	>=8/152	>=4	>=8	>=8	<=2	0.12	1	2

LNZ, linezolid; PEN, penicillin G; OXA, oxacillin; CHL, chloramphenicol; CLI, clindamycin; ERY, erythromycin; GEN, gentamicin; SXT, trimethoprim/sulfamethoxazole; CIP, ciprofloxacin; LVX,levofloxacin; RIF, rifampin; TCY, tetracycline; TGC,tigecycline; VAN,vancomycin; TEC, teicoplanin. pos, positive; neg, negative

### Molecular characteristics and the mechanisms of linezolid resistance

Seven of the eight *S. epidermidis* isolates belonging to the same clone, ST22, had two point mutations (T2504A and C2534T) in domain V of the 23 S rRNA gene, and exhibited L101V, G152D, and D159Y changes in the amino acid sequences of the L3 protein and a N158S change in the L4 protein. No *cfr* genes were detected in the seven ST22-type *S. epidermidis* isolates. However, another ST2-type *S. epidermidis* isolate with positive carriage of the *cfr* gene but no point mutation in domain V of the 23 S rRNA gene was found(Table 3).

**Table 3:** Characteristics and drug resistance mechanisms of linezolid-resistant MRCoNS

Isolate	MLST	*cfr* gene	**23S rRNA mutations**	**Ribosomal protein mutation**			Other resistance genes
				L3	L4	L22	
*S.epidermidis*-1	ST22	−	T2504A C2534T	L101V, G152D, D159Y	N158S	−	*mecA, blaZ, aac(6')-aph(2''), ant(6)-Ia, aph(3')-III, qacA, fusB, fosB, msr(A),mph(C)*
*S.epidermidis*-2	ST22	−	T2504A C2534T	L101V, G152D, D159Y	N158S	−	*mecA, blaZ, aac(6')-aph(2''), ant(6)-Ia, aph(3')-III, fusB, qacA, mecA, blaZ, fosB, msr(A), mph(C)*
*S.epidermidis*-3	ST22	−	T2504A C2534T	L101V, G152D, D159Y	N158S	−	*mecA, blaZ, aac(6')-aph(2''), ant(6)-Ia, aph(3')-III, qacA, fosB, fusB, msr(A), mph(C)*
*S.epidermidis-*4	ST22	−	T2504A C2534T	L101V, G152D, D159Y	N158S	−	*mecA, blaZ, aac(6')-aph(2''), ant(6)-Ia, aph(3')-III, qacA, fosB, fusB, msr(A), mph(C)*
*S.epidermidis*-5	ST22	−	T2504A C2534T	L101V, G152D, D159Y	N158S	−	*mecA, blaZ, aac(6')-aph(2''), ant(6)-Ia, aph(3')-III, qacA, fosB, fusB, msr(A), mph(C)*
*S.epidermidis*-6	ST22	−	T2504A C2534T	L101V, G152D, D159Y	N158S	−	*mecA, blaZ, aac(6')-aph(2''), ant(6)-Ia, aph(3')-III, qacA, fosB, fusB, msr(A), mph(C)*
*S.epidermidis*-7	ST2	+	−	L101V	−	−	*mecA, blaZ, aac(6')-aph(2''), qacA, fosB, erm(T), mupA*,
*S.epidermidis*-8	ST22	−	T2504A C2534T	L101V, G152D, D159Y	N158S	−	*mecA, blaZ, aac(6')-aph(2''), ant(6)-Ia, aph(3')-III, qacA, fosB, fusB, msr(A),mph(C)*
*S.cohnii-*9	N/D	+	−	S158Y, D159Y	−	−	*mecA, qacA, fosB, erm(C), mupA, tet(K), dfrG*
*S.cohnii*-10	N/D	−	−	S158Y, D159Y	−	−	*mecA, aac(6')-aph(2''), qacA, fosB, erm(C), mupA*
*S.cohnii*-11	N/D	+	−	−	−	−	*mecA, aac(6')-aph(2''), qacA, erm(C), mupA, dfrG*,
*S.cohnii*-12	N/D	+	−	S158Y, D159Y	−	−	*mecA, aac(6')-aph(2''), qacA, fosB, erm(C), mupA, tet(K)*
*S.cohnii*-13	N/D	+	−	S158Y, D159Y	−	−	*mecA, aac(6')-aph(2''), qacA, erm(C), mupA, tet(K), dfrG*
*S.cohnii*-14	N/D	+	−	S158Y, D159Y	−	−	*mecA, aac(6')-aph(2''),qacA, fosB, fexA, mupA, tet(K), dfrG*
*S.hominis*-15	ST2	+	−	−	−	−	*mecA, blaZ, aadD, aac(6')-aph(2''), bleO, qacA, fosB, fusC, erm(C), mecA, blaZ, mupA, tet(K), dfrG*
*S.hominis*-16	ST1	−	G2576T	−	−	−	*mecA, blaZ, aadD, aac(6')-aph(2''), bleO, qacA, lnu(A), mupA*
*S.hominis*-17	ST1	−	G2576T	−	−	−	*mecA, blaZ, aadD, aac(6')-aph(2''), bleO, qacA, lnu(A), mupA*
*S.hominis*-18	ST1	−	G2576T	−	−	−	*mecA, blaZ, aadD, aac(6')-aph(2''), bleO, qacA, lnu(A), mupA*,
*S.hominis*-19	ST1	+	−	−	−	−	*mecA, blaZ, aac(6')-aph(2''), qacA, fusC, erm(C), mupA*
*S.hominis*-20	ST1	+	−	−	−	−	*mecA, blaZ, aac(6')-aph(2''), qacA, erm(C), lnu(A), mupA*
*S.hominis*-21	ST85	+	−	−	T124I	−	*mecA, aadD, aac(6')-aph(2''), bleO, qacA, qacB, msr(A), lnu(A)*
*S.hominis*-22	ST85	+	−	−	−	−	*mecA, aac(6')-aph(2''), qacA, erm(C), mupA*
*S.hominis*-23	ST2	+	−	V154L,M156T	−	−	*mecA, blaZ, aadD, aac(6')-aph(2''), ant(6)-Ia, aph(3')-III, qacA, msr(A), lsa(B), mph(C), mupA, tet(K)*
*S. capitis-*24	N/D	+	G2576T C2104T	M156T	I188S	−	*mecA, blaZ, aadD, aac(6')-aph(2''), ant(9)-Ia, bleO, qacA, erm(A)*
*S. capitis*-25	N/D	+	G2576T C2104T	M156T	I188S	−	*mecA, blaZ, aadD, aac(6')-aph(2''), ant(9)-Ia, bleO, qacA, erm(A)*
*S. capitis*-26	N/D	−	G2576T C2104T	M156T	I188S	−	*mecA, blaZ, aadD, aac(6')-aph(2''), ant(9)-Ia, bleO, qacA, erm(A)*
*S. capitis*-27	N/D	−	G2576T C2104T	−	I188S	−	*mecA, blaZ, aadD, aac(6')-aph(2''), ant(9)-Ia, bleO, qacA, erm(A)*
*S. capitis*-28	N/D	+	G2576T C2104T	M156T	I188S	−	*mecA, blaZ, aadD, aac(6')-aph(2''), ant(9)-Ia, bleO, qacA, erm(A)*
*S. capitis*-29	N/D	+	G2576T C2104T	M156T	I188S	−	*mecA, blaZ, aadD, aac(6')-aph(2''), ant(9)-Ia, bleO, qacA, fosB, erm(A)*
*S. capitis-*30	N/D	+	G2576T C2104T	V154L	I188S	−	*mecA, blaZ, aadD, aac(6')-aph(2''), ant(9)-Ia, bleO, qacA, erm(A)*
*S. capitis*-31	N/D	+	G2576T C2104T	M156T	I188S	−	*mecA, blaZ, aadD, aac(6')-aph(2''), ant(9)-Ia, bleO, qacA, erm(A)*
*S. capitis-*32	N/D	−	G2576T C2104T	−	I188S	−	*mecA, blaZ, aadD, aac(6')-aph(2''), ant(9)-Ia, bleO, qacA, fusB, erm(A)*
*S. capitis*-33	N/D	+	G2576T C2104T	M156T	I188S	−	*mecA, blaZ, aadD, aac(6')-aph(2''), ant(9)-Ia, bleO, qacA, erm(A)*
*S. capitis*-34	N/D	+	G2576T C2104T	M156T	I188S	−	*mecA, blaZ, aadD, aac(6')-aph(2''), ant(9)-Ia, bleO, qacA, erm(A)*
*S. capitis*-35	N/D	−	G2576T C2104T	−	I188S	−	*mecA, blaZ, aadD, aac(6')-aph(2''), ant(9)-Ia, bleO, qacA, fusB, erm(A)*
*S. capitis*-36	N/D	−	G2576T C2104T	−	I188S	−	*mecA, blaZ, aadD, aac(6')-aph(2''), ant(9)-Ia, bleO, qacA, fusB, erm(A)*
*S. haemolyticus*-37	ST1	+	−	R138V	−	−	*mecA, aadD, aac(6')-aph(2''), msr(A), lsa(B), mph(C), vga(A)LC, mupA*

N/D, not determined

**Fig. 1:**
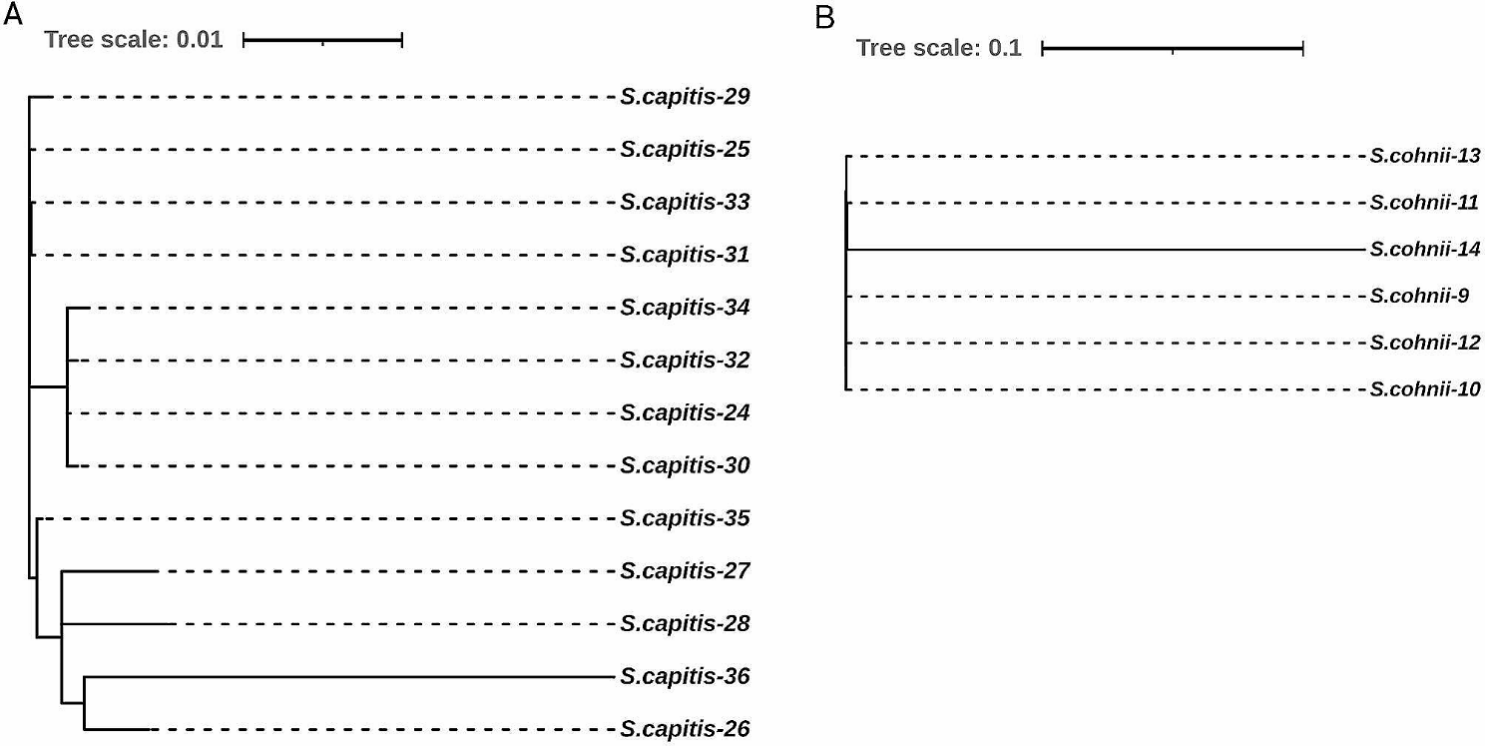
Phylogenetic tree (based on the SNP strategy) analysis of *S. capitis* isolates and *S. cohnii* isolates. (**A**) *S. capitis* isolates. (**B**) *S. cohnii* isolates

The six *S. cohnii* isolates were found to belong to the same clone by phylogenetic analysis (Fig. 1) and carried the *cfr* gene, as well as had S158Y and D159Y changes in the L3 protein. No mutation in domain V of the 23 S rRNA gene was detected in any of the *S. cohnii* isolates.

The nine *S. hominis* isolates were divided into 3 distinct clones: ST1 (*n* = 5), ST2 (*n* = 2) and ST85 (*n* = 2). A novel ST type, ST85, was found for the first time in this study. The *cfr* gene was identified among six *S. hominis* isolates, while the other three *S. hominis* isolates without the *cfr* gene had a 23 S rRNA G2576T mutation.

In the present study, clone spread was found among those *S. capitis* isolates by phylogenetic analysis (Fig. 1), and G2576T and C2104T 23 S rRNA mutations were identified. Additionally, 8 *S. capitis* isolates (61.5%) harbored the *cfr* gene. Furthermore, M156T and I188S changes were identified in the L3 and L4 proteins in most *S. capitis* strains. *S. haemolyticus*-37 harbored the *cfr* gene and had an additional R138V change in the L3 protein.

No mutation in the L22 protein was detected in any of the linezolid-resistant MRCoNS isolates in this study.

In addition to linezolid-related resistance genes, other resistance genes were also detected in our study, and the *mecA* gene was detected in all linezolid-resistant MRCoNS isolates (Table 3).

## Discussion

In the present study, 37 linezolid-resistant MRCoNS isolates were obtained from a large tertiary teaching hospital from December 2019 to March 2023. In this hospital linezolid-resistant MRCoNS strains were first found in 2016; however, the detection rate of linezolid-resistant MRCoNS strains has steadily increased in recent years. Linezolid is an important alternative for the management of MRCoNS infections. The rapid emergence of linezolid-resistant MRCoNS is alarming and requires ongoing surveillance. Previous studies have indicated that linezolid administration is a significant risk factor for linezolid-resistant gram-positive cocci during hospital outbreaks [22, 23]. Our data showed that 13 patients (36.1%) had received prior linezolid therapy, thus, we speculated that increasing selective pressure most likely contributed to drug resistance. In addition, all 37 isolates were resistant to multiple antibiotics, and various resistance genes were detected, which indicated that the treatment options were limited.

Multilocus sequence typing indicated that ST22 was the dominant clone among the *S. epidermidis* isolates. The results of the present study were consistent with studies on the *S. epidermidis* lineage in Greece and Spain [24, 25] and different from the findings in Germany and France (ST2) [26, 27]. ST2, ST5, and ST22 are clustered into the CC5 clone, which is the most prevalent clonal complex among the nosocomial *S. epidermidis* population according to the literature [28]. Although linezolid-resistant CoNS strains have been reported sporadically worldwide, *S. hominis* pathogens that are resistant to linezolid are uncommon. Nine *S. hominis* strains were isolated in the present study, ST1 was the predominant clone, and ST85, a novel ST type, was found for the first time in our study.

MLST typing of *S. epidermidis* and *S. hominis* as well as phylogenetic analysis of *S. capitis* and *S. cohnii* suggested the transmission of resistant clones from patient to patient and clonal spread within the intensive care unit in our hospital.

The primary causes of linezolid resistance among *staphylococci* include modification of the target site of 23 S rRNA, acquisition of the *cfr* gene, and mutations of the ribosomal proteins L3 and L4 [11–16]. G2576T was the most frequently detected mutation in domain V of the 23 S rRNA gene, In addition, mutations in 23 S rRNA at positions G2534T, G2603T, T2504A and T2500A are also associated with reduced linezolid susceptibility [11–13]. In the present study, three ST1-type *S. hominis* isolates had a G2576T mutation, and thirteen *S. capitis* isolates coharbored G2576T and C2104T mutations, similar results have also been reported in previous studies [29, 30]. Furthermore, T2504A and C2534T mutations were found among seven ST22-type *S. epidermidis* isolates, yet no mutation in domain V of the 23 S rRNA gene was detected in *S. cohnii* or *S. haemolyticus*.

Acquisition of the *cfr* gene constitutes the second mechanism of target site mutation in staphylococci, and the *cfr* gene is usually located on a plasmid and confers resistance to linezolid [15, 27]. In the present study, the *cfr* gene was detected in one *S. epidermidis*, one *S. haemolyticus*, five *S. cohnii*, six *S. hominis* and eight *S. capitis strains*. Interestingly, the *cfr* gene was found in ST2-type *S. epidermidis* but not in ST22-type *S. epidermidis*, and the findings indicated that strains of distinct clones had diverse mechanisms of linezolid resistance.

Mutations of the ribosomal proteins L3, L4 and L22 were also analyzed in this study, and *S. epidermidis* and *S. capitis* isolates showed a series of alterations in the ribosomal proteins L3 and L4. Notably, the M156T mutation in the L3 protein and the I188S mutation in the L4 protein were also detected among *S. capitis* isolates for the first time. Mutations in the ribosomal L3 and L4 proteins were not detected among the seven *S.hominis* isolates. Notably, our data demonstrated that the mechanism of linezolid resistance in *S. epidermidis* and *S. capitis* was complex and involved simultaneous acquisition of the MDR gene *cfr*, as well as mutations of the target site 23 S rRNA and ribosomal proteins L3 and L4, and the linezolid MIC was greater than that in *S. cohnii* and *S. hominis*. These multiple resistance mechanisms could contribute to more high-level linezolid resistance than a single resistance mechanism.

In conclusion, there has been an increase in the prevalence of linezolid resistance among CoNS in our hospital’s intensive care units in recent years. Additionally, many of the isolates were clonally related, suggesting the intrahospital dissemination of resistant clones. Resistance is related to the presence of the *cfr* gene, a point mutation in the V domain of the 23 S rRNA gene and/or a mutation in the ribosomal L3 and L4 proteins, and multiple drug resistance mechanisms often coexist. Notably, distinct CoNS have different mechanisms of linezolid resistance. Taken together, these findings on the spread of linezolid-resistant CoNS in our setting highlight the importance of monitoring linezolid resistance in MRCoNS. Strict control measures should be taken to prevent further dissemination, and the relevant use of antibiotics needs to be emphasized.

## Data Availability

The sequence data generated in this study have been submitted to the NCBI BioProject database (https://www.ncbi.nlm.nih.gov/bioproject/) under accession numbers PRJNA1077915.

## Abbreviations

CoNS: coagulase-negative *staphylococci*
MRCoNS: methicillin-resistant coagulase-negative *staphylococci*
MLST: multilocus sequence typing
CARSS: China Antimicrobial Resistance Surveillance System
CLSI: Clinical and Laboratory Standards Institute
WGS: whole-genome sequencing
